# Exploring Antiviral Drugs on Monolayer Black Phosphorene: Atomistic Theory and Explainable Machine Learning-Assisted Platform

**DOI:** 10.3390/ijms25094897

**Published:** 2024-04-30

**Authors:** Slimane Laref, Fouzi Harrou, Ying Sun, Xin Gao, Takashi Gojobori

**Affiliations:** 1 Computational Bioscience Research Center (CBRC), King Abdullah University of Science and Technology (KAUST), Thuwal 23955-6900, Saudi Arabia; xin.gao@kaust.edu.sa (X.G.); takashi.gojobori@kaust.edu.sa (T.G.); 2 Computer, Electrical and Mathematical Sciences and Engineering (CEMSE) Division, King Abdullah University of Science and Technology (KAUST), Thuwal 23955-6900, Saudi Arabia; ying.sun@kaust.edu.sa

**Keywords:** ensemble learning, DFT, inhibitor, black phosphorus, thermodynamic, molecular states, drug vehicles

## Abstract

Favipiravir (FP) and ebselen (EB) belong to a diverse class of antiviral drugs known for their significant efficacy in treating various viral infections. Utilizing molecular dynamics (MD) simulations, machine learning, and van der Waals density functional theory, we accurately elucidate the binding properties of these antiviral drugs on a phosphorene single-layer. To further investigate these characteristics, this study employs four distinct machine learning models—Random Forest, Gradient Boosting, XGBoost, and CatBoost. The Hamiltonian of antiviral molecules within a monolayer of phosphorene is appropriately trained. The key aspect of utilizing machine learning (ML) in drug design revolves around training models that are efficient and precise in approximating density functional theory (DFT). Furthermore, the study employs SHAP (SHapley Additive exPlanations) to elucidate model predictions, providing insights into the contribution of each feature. To explore the interaction characteristics and thermodynamic properties of the hybrid drug, we employ molecular dynamics and DFT calculations in a vacuum interface. Our findings suggest that this functionalized 2D complex exhibits robust thermostability, indicating its potential as an effective and enabled entity. The observed variations in free energy at different surface charges and temperatures suggest the adsorption potential of FP and EB molecules from the surrounding environment.

## 1. Introduction

The rapid advancement of clinical biomedicine and nanobiotechnology has served as a catalyst for the emergence of a wide range of inorganic nanoparticles. These nanoparticles offer diverse pattern modalities, presenting potential alternatives for addressing diseases through synergistic therapy, particularly in the treatment of metastatic conditions [1,2,3]. The remarkable and distinct properties of two-dimensional (2D) materials have garnered significant attention [4,5,6]. Building upon the success of graphene, a multitude of 2D materials have been harnessed for a wide array of applications related to properties, encompassing energy, environmental science, catalysis, physics, and biomedicine [7,8,9,10,11,12]. For instance, materials like transition metal dichalcogenides (TMDs), nitrides and carbonitrides (MXenes), and hexagonal boron nitride (h-BN), along with their derivatives, have been successfully synthesized in monolayer and few-layer forms. These materials are characterized by weak van der Waals interlayer bonding and strong in-plane covalent bonding [13,14,15]. Consequently, there is considerable ongoing research focused on exploring the biological behavior of these 2D Xenes nanomaterials to assess their biosafety and biocompatibility, including aspects such as biodegradability, cytotoxicity, and the presence of any toxic derivatives [16]. The broad spectrum of potential applications and clinical integration of 2D MXenes systems in the context of biomedical drug therapy mandates a meticulous examination of their biodegradability [17] and toxicity. Simultaneously, it necessitates a thorough comprehension of the intricate biochemical interactions occurring between drug molecules and 2D nanomaterials at their interfacial regions. Of particular significance is the understanding of molecular orientations within multi-layered materials, as it enables the development of biologically compatible materials for gene silencing, dispersing complexes, hybrid pharmaceutical ingredients, and biomedical sensing.

In the current investigation, prompted by recent advancements in the synthesis of two-dimensional (2D) nanomaterials and their promising application in the development of anti-tumor pharmaceuticals [18,19,20], a comprehensive research endeavor is undertaken. The primary objective of this study is to explore the feasibility of utilizing 2D materials as nanocarriers for drug delivery purposes. Two noteworthy pharmaceutical candidates considered in this context are ebselen (EB) and favipiravir (FP) at the molecular level [21,22]. The utilization of machine learning methods in the fields of nanomaterials and pharmaceutical technologies has become a subject of considerable interest among researchers in recent years. Numerous investigations have employed backpropagated neural networks to effectively model diverse systems [23,24]. Examples of such systems include Darcy–Forchheimer slip flow models for nanomaterials and ferrofluids [25], non-Fourier heat flux models for transient heat exchange [26], and ternary nanofluid flow between parallel plates [27]. To evaluate the accuracy of these models, we obtained reference datasets through the homotopy analysis method, and numerical methods were employed. The present study encompasses an analysis of the influence of non-dimensional parameters on the studied systems. This research employs an integrated approach, encompassing accelerated molecular dynamics, machine learning techniques, and quantum chemistry based on first-principles density functional theory (DFT). The primary focus of this investigation is to provide a succinct overview of the structural stability and thermodynamic properties of both FP and EB when functionalized within a typical phosphorene 2D monolayer. The examination of chemical interactions between FP, EB, and 2D black-phosphorus materials is undertaken, taking into account the vacuum model. Furthermore, the study delves into the quantification of free energy changes associated with each step of this process. This approach enables us to demonstrate the capacity to modulate the release ratio of antiviral molecules onto the phosphorene nanosheet by manipulating temperature and physicochemical parameters.

This study provides valuable insights into the promising application of emerging two-dimensional (2D) materials as novel nanocarriers for antiviral drug therapy. These atomistic findings open the door to the parametrization of multiscale methodologies, such as contemporary machine learning based on electronic theories, continuum models, Monte Carlo simulations, and coarse-grained methods, to address the interplay between the interaction of drugs/2D monolayers with the cell membrane as a function of their morphology. These methodologies can be employed to comprehensively investigate the intricate interactions between drugs and the 2D monolayer with the cell membrane, considering variations in their morphology. Additionally, our research conducts a thorough examination of the effectiveness of four ensemble learning models in expediting the prediction of van der Waals energy, non-bond energy, and total potential energy associated with ebselen (EB), favipiravir (FP), and their combination (FP_EB). Specifically, we assess the performance of Random Forest, Gradient Boosting, XGBoost, and CatBoost in this context. The rationale behind employing multiple models is to facilitate a comprehensive comparison and evaluation of their respective abilities to predict the behavior of antiviral molecules within the 2D monolayer with precision. The results highlight the efficacy of the four ensemble learning models. Their superior performance can be attributed to their unique capacity to amalgamate multiple weak learners, achieving robust prediction accuracy. By effectively harnessing the complementary strengths of individual learners, ensemble models exhibit enhanced resilience to noise, outliers, and model biases, resulting in superior performance across a wide range of datasets and predictive tasks. The successful training of efficient and accurate models to approximate density functional theory (DFT) marks a crucial milestone in leveraging machine learning approaches to gain deeper insights into the fostering of novel drugs. Furthermore, the study employs SHAP (SHapley Additive exPlanations) to elucidate model predictions, providing insights into the contribution of each feature.

The remaining sections of the paper are organized as follows. Section 2 presents the statistical analysis of the data used in the study and discusses the prediction results of the three energies, MDS results, and density functional calculations. Section 3 provides a brief overview of molecular dynamics simulations, the four ensemble machine learning models considered, and the proposed prediction framework. Finally, Section 4 concludes the paper.

## 2. Results and Discussion

### 2.1. Data Description and Analysis

Molecular dynamics represents a potent approach facilitating interactions among atoms in systems based on specified parameters like temperature, time, pressure, and other environmental conditions. Hence, integrating molecular dynamics methods with ensemble learning and other quantum techniques is a promising research direction. The central objective of this study is to optimize the terms related to total energy, van der Waals energy, and non-bond energy obtained from molecular dynamics simulations. Datasets are created utilizing the COMPASS III [28] force field for molecular dynamics simulations, focusing on small antiviral molecules on a BP single layer [29]. Employing the ensemble learning models, the Hamiltonian and interaction energy are trained under periodic boundary conditions (PBCs) for polycyclic molecules positioned atop a phosphorene monolayer. The ultimate objective is to train efficient and accurate models for approximating density functional theory (DFT), culminating in the utilization of ensemble learning to assist in the design of novel drugs.

This section aims to thoroughly assess the predictive accuracy of molecular dynamics (MD) simulations and machine learning models in estimating the total energy, van der Waals energy, and non-bond energy. The evaluation was carried out using three separate datasets: EB, FP, and FP_EB. Van der Waals energy represents the transient attractive or repulsive forces between molecules, while total potential energy encompasses all interatomic interactions within a system. Non-bond energy, on the other hand, quantifies interactions beyond covalent or ionic bonds, such as van der Waals forces and electrostatic interactions. Accurately predicting these energies using machine learning approaches offers invaluable insights into molecular stability, binding affinity, and system behavior across various scientific domains. These predictions aid in drug discovery, materials design, and biological studies by providing fast and precise estimations of molecular properties, facilitating the identification of promising candidates and the optimization of molecular structures with enhanced properties.

**Figure 1:**
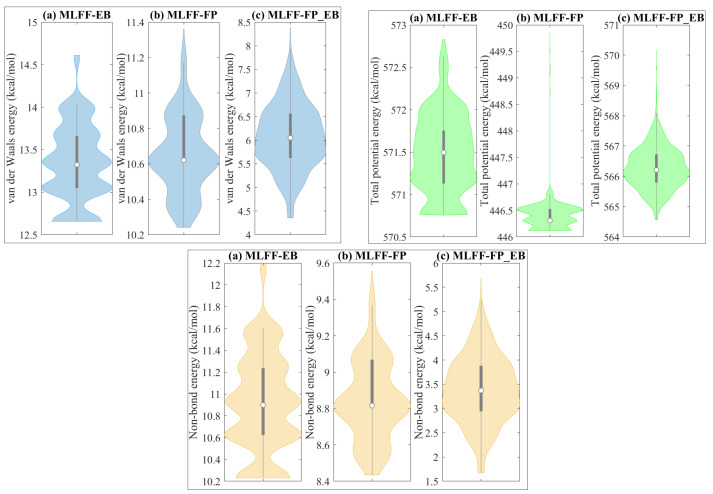
The data distribution for van der Waals energy, total potential energy, and non-bond energy for three distinct cases: (**a**) EB, (**b**) FP, and (**c**) FP_EB.

Figure 1 illustrates the data distribution through violin plots of van der Waals energy, total potential energy, and non-bond energy under EB, FP, and FP_EB.Violin plots offer a combination of a box plot and a kernel density plot, allowing for an easy comparison of the distribution shape, spread, and central tendency across multiple datasets. They effectively convey the variability and density of the data, enabling the identification of patterns, outliers, and potential trends within each energy parameter across different simulation conditions. We observe that these variables deviate from a Gaussian (normal) distribution. This finding poses a difficulty for conventional prediction techniques, like principal component regression, which assumes Gaussian data distributions. Hence, there is a significant way to apply machine learning models that are not dependent on particular data distributions. From the left panel of Figure 1, it can be noted that the van der Waals energy decreases under FP_EB compared to EB and FP. This observation suggests a distinct impact of the combined FP_EB case on the van der Waals energy compared to the individual favipiravir and ebselen standalone cases. In the middle panel of Figure 1, it is apparent that the total potential energy decreases in the FP adsorbed state compared to both EB and FP_EB scenarios. This can be attributed to the unique chemical properties and interactions of the favipiravir molecule. FP can potentially induce changes in the overall molecular configuration or alter the bonding interactions within the system, resulting in a decrease in total potential energy. Variations in molecular size, shape, and electronic structure between FP and the other molecules considered can contribute to this phenomenon. Nevertheless, further in-depth analysis and investigation are imperative to fully elucidate the underlying reasons for this observed disparity. From the left panel of Figure 1, it is clear that the non-bond energy decreases in the FP_EB compared to EB and FP. This indicates that combining favipiravir (FP) and ebselen (EB) affects the non-bond energy differently than using them individually. The alteration observed can be attributed to the distinctive chemical interactions between FP and EB within the BP monolayer, as detailed in reference [29].

**Figure 2:**
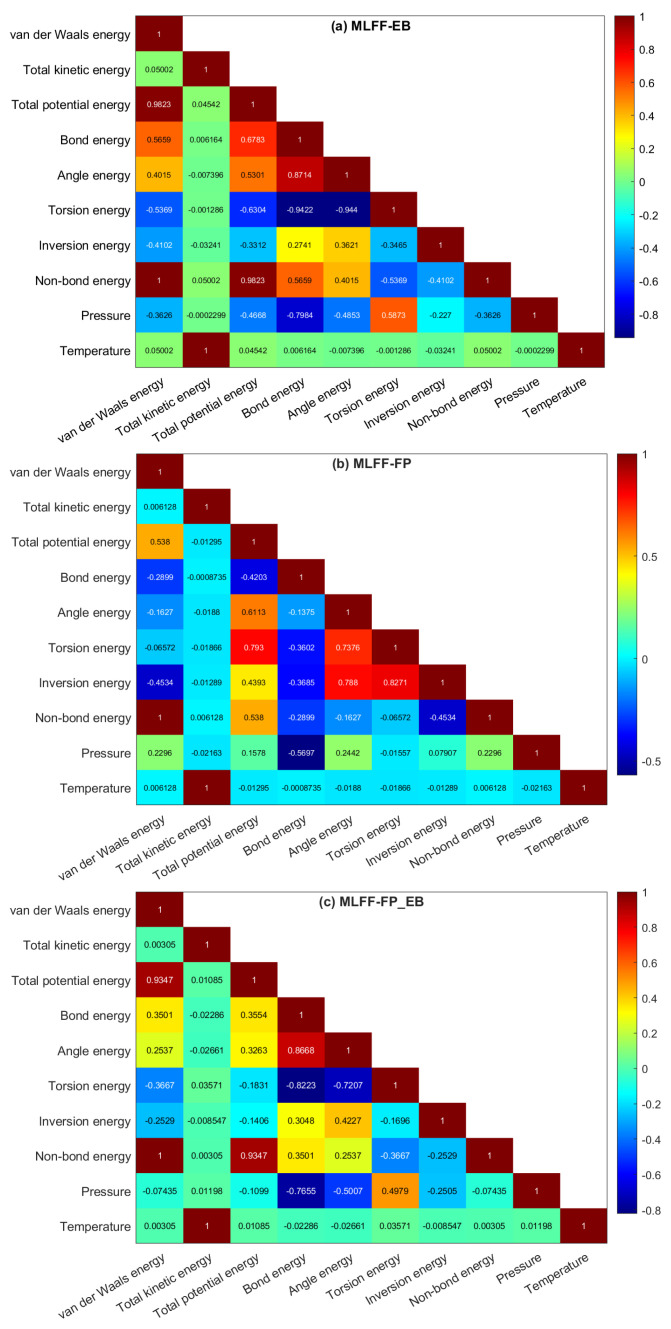
Heatmap illustrating the correlation matrix: (**a**) EB, (**b**) FP, and (**c**) FP_EB.

Figure 2 illustrates the pairwise correlation coefficients among the multivariate data of antiviral drugs on the BP monolayer, EB, FP, and FP_EB. The correlation matrix provides insight into relationships and dependencies between variables, aiding in feature selection, dimensionality reduction, and understanding complex datasets. From Figure 2a–c, We note a strong correlation between temperature and kinetic energy, which is expected, as temperature directly influences the movement and speed of particles in a system. However, the lack of correlation between temperature and other variables suggests that temperature variations do not significantly affect potential energy, non-bond energy, or total energy in this context. This observation can indicate that changes in temperature primarily impact kinetic energy, while the other energy components remain relatively unaffected. We observe strong correlation values between van der Waals energy and total potential energy, with coefficients of 0.9823 and 0.9347 under EB and FP_EB, respectively. However, under FP, the correlation decreases to a moderate level, with a coefficient of 0.538. This decrease in correlation suggests that the relationship between van der Waals energy and total potential energy is less pronounced under the FP scenario compared to the other two. Additional insights are discussed in the DFT section to elucidate the factors contributing to this difference and their implications for molecular dynamics simulations and drug design. We notice strong correlation values between angle energy and bond energy, with coefficients of 0.8714 and 0.8668 under EB and FP_EB, respectively. However, under FP, the correlation decreases to a low level, with a coefficient of −1.1375. This decrease in correlation suggests that the relationship between angle energy and bond energy is significantly weaker under the FP scenario compared to EB and FP_EB. It indicates potential differences in the interactions between these energy components in the context of FP, which can be attributed to specific features or parameters of the FP model. In the calculations, the entire cell, along with its external pressure, remains fixed. Under EB and FP_EB, this external pressure exhibits a moderate negative correlation with bonding energy and angle energy while showing a positive moderate correlation with torsion energy (Figure 2a–c). However, under FP, the correlation between pressure and bonding energy and torsion energy decreases to a low level. This change suggests that the relationship between pressure and these energy components is significantly weaker in the FP scenario compared to EB and FP_EB. In the subsequent section, we delve deeper into energy decomposition to thoroughly explore the factors underlying this variation and their importance for computational simulations in the fields of materials science and chemistry.

**Figure 3:**
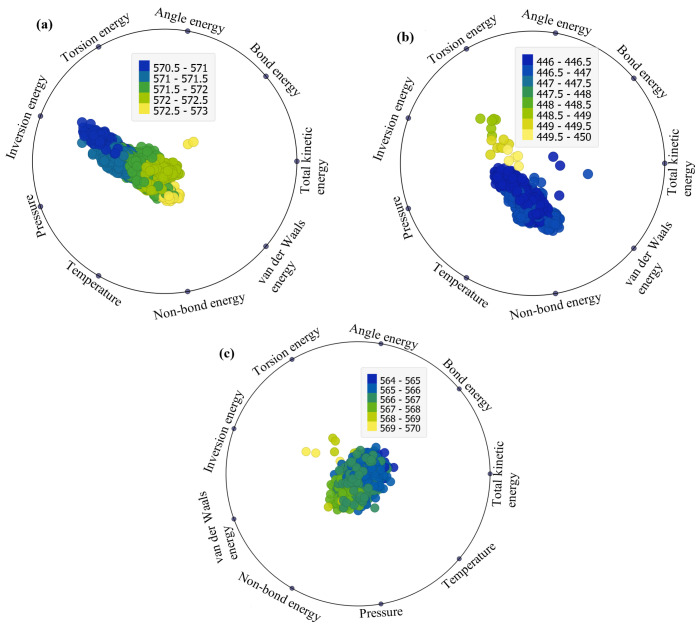
RadViz plots of the factors impacting the total potential energy (TPE) under (**a**) EB, (**b**) FP, and (**c**) FP_EB systems.

The RadViz visualizations depicted in Figure 3a–c offer a comprehensive representation of the factors impacting total potential energy across the three considered structures (EB, FP, and FP_EB). Originally designed for classifying DNA sequences, RadViz positions observations on a unit circle, connecting each point to anchor points representing the variables of interest [30]. The connections are established through virtual springs, with each observation’s position influenced by the relative strengths of these springs. Equilibrium positions are reached where the system of spring forces is balanced [31]. Notably, observations in the circle’s center have roughly equal dimensional values, while those with significantly larger coordinate values are closer to the corresponding dimensional anchors. This method provides an intuitive and insightful way to explore and interpret the relationships and patterns within multi-dimensional datasets in a reduced 2D space. RadViz facilitates the easy interpretation of relationships and patterns between variables, allowing for the identification of clusters, outliers, and trends within the data. Overall, the RadViz plots offer valuable insights into our dataset by visually depicting the relationships between different variables and their impact on the target variables, which include total potential energy, van der Waals energy, and non-bond energy under the investigated structures (EB, FP, and FP_EB). By examining the directional attractions and shifts in the plots, we can discern patterns and trends in how specific variables influence these energy parameters across varying scenarios. This visual representation aids in identifying the key factors driving variations in these energy parameters.

**Figure 4:**
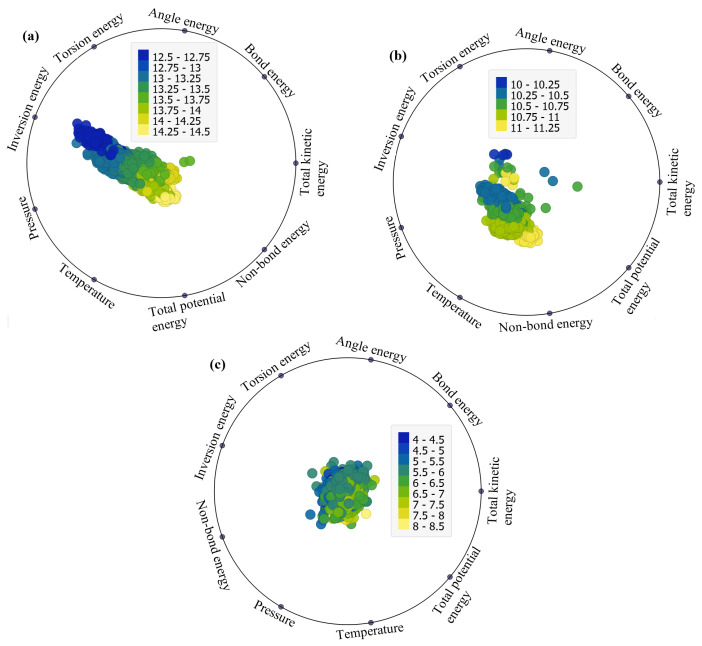
RadViz plots of the factors impacting the van der Waals energy under (**a**) EB, (**b**) FP, and (**c**) FP_EB.

**Figure 5:**
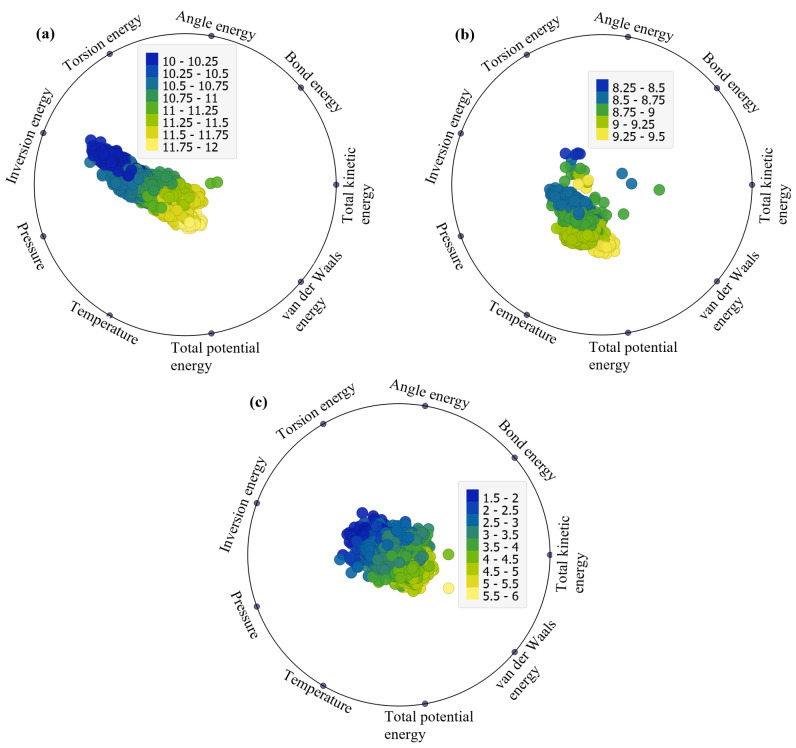
RadViz plots of the factors impacting the non-bond energy under (**a**) EB, (**b**) FP, and (**c**) FP_EB.

Figure 3 presents RadViz plots illustrating the factors influencing total potential energy (TPE) under different adsorption states: (a) EB, (b) FP, and (c) FP_EB configurations. From Figure 3a, it is evident that under EB, TPE is significantly impacted by inversion energy and van der Waals energy. Additionally, non-bond energy, van der Waals energy, and inversion energy play crucial roles. However, under FP_EB, the data appear centered, with no noticeable influence from specific factors (Figure 3c). The prominence of inversion energy and van der Waals energy influencing TPE under EB conditions suggests that these factors play a significant role in determining the total potential energy of the system. Inversion energy relates to the energy associated with the inversion of bonds and structural deformations within the molecular system, while van der Waals energy represents the attractive, repulsive forces between molecules and surfaces. Therefore, the observed influence indicates that changes in molecular structure and intermolecular interactions, particularly those related to bond deformations and van der Waals forces, have a substantial impact on the overall potential energy of the system under EB conditions. On the other hand, under FP_EB, where both favipiravir (FP) and ebselen (EB) are present, the data appear centered with no clear influence from specific factors. This can be attributed to the interactions between FP and EB molecules, which may result in a balanced or neutralizing effect on the overall energy contributions from individual factors. When both FP and EB are present together, their combined interactions can potentially offset or counteract the individual effects observed under separate FP and EB conditions. As a result, the data distribution appears centered, indicating a more balanced or stabilized energy landscape, where the influence of specific factors is less pronounced. This suggests a synergistic or cooperative effect between FP and EB molecules, leading to a more uniform distribution of energy contributions across different factors. Similarly, from Figure 4 and Figure 5, the factors impacting the van der Waals energy and non-bond energy can be visually identified.

This study employs the XGBoost algorithm to determine the most influential variables affecting the computed energies, including total potential energy (TPE), van der Waals energy, and non-bond energy. XGBoost offers a feature importance ranking mechanism, assigning scores to each input variable based on their impact on the model’s predictive performance. By analyzing these scores, we can identify the key variables that significantly contribute to the variability in the computed Hamiltonian. This allows us to prioritize and focus on the most relevant features, providing valuable insights into the underlying factors driving the energy dynamics in our system.

**Figure 6:**
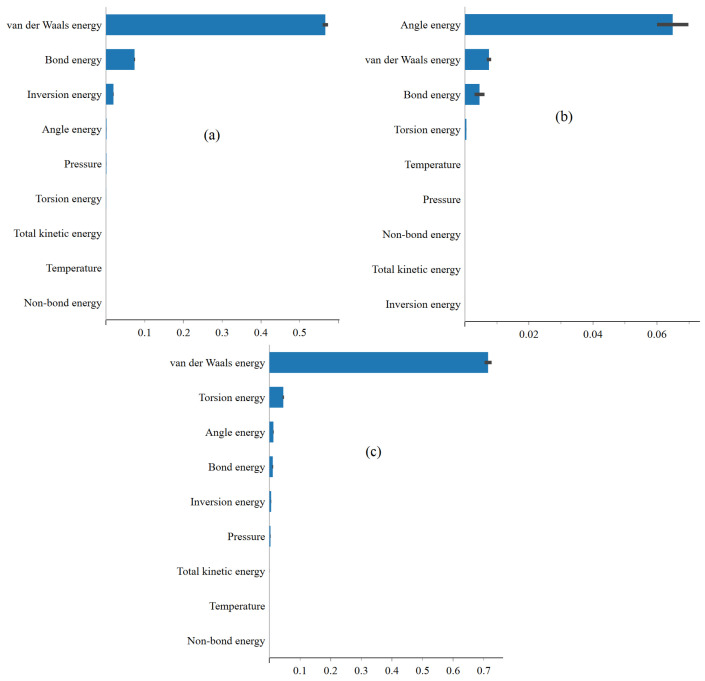
Feature importance identification using XGBoost for predicting the total potential energy (TPE) under (**a**) EB, (**b**) FP, and (**c**) FP_EB systems.

**Figure 7:**
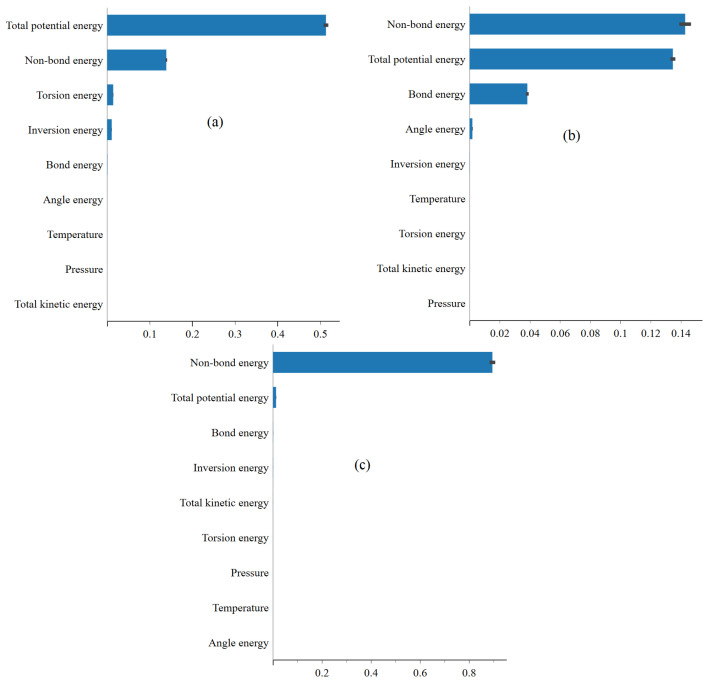
Feature importance identification using XGBoost when predicting the van der Waals energy under (**a**) EB, (**b**) FP, and (**c**) FP_EB.

**Figure 8:**
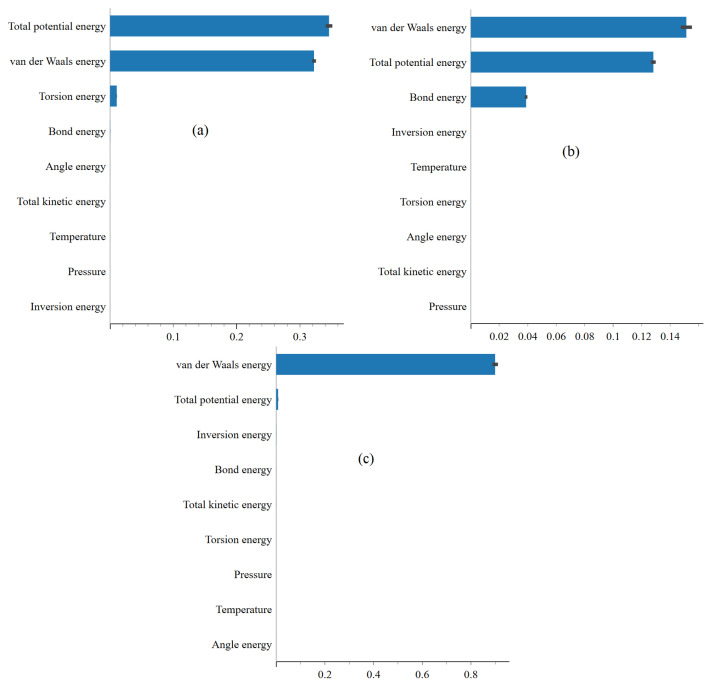
Feature importance identification using XGBoost when predicting the non-bond energy under (**a**) EB, (**b**) FP, and (**c**) FP_EB.

In Figure 6a–c, the feature importance plots highlight the significance of energy terms, such as van der Waals, bond, and angle energies, in predicting total potential energy landscapes. The predominance of van der Waals and angular energies underscores their pivotal role in shaping molecular stability, particularly concerning structural flexibility and conformational preferences. This result underscores the importance of understanding molecular characteristics and processes driving the behavior of the system. Furthermore, favorable physisorption of FP, EB, and FP_EB molecules onto the BP surface is observed, with enhanced interactions noted for the hybrid FP_EB system. According to [29], the analysis of electronic properties reveals alterations in frontier molecular orbitals and energy gaps upon adsorption, alongside charge redistribution and electron transfer at the interface between the molecules and BP monolayer. Combining FP and EB on a BP sheet is suggested as a potential strategy for creating an effective drug delivery system with controllable temperature-dependent drug release mechanisms, offering promising applications in targeted drug delivery and hyperthermia treatment. Lastly, the representation of the total potential energy feature importance identification using XGBoost enriches our understanding of the interactions between antiviral molecules and the single-layer BP surface, paving the way for designing and developing innovative drug delivery systems tailored to specific therapeutic requirements. Figure 7 and Figure 8 respectively display the feature importance identified using XGBoost for predicting the van der Waals energy and non-bond energy under (a) EB, (b) FP, and (c) FP_EB. Similarly, we can visually identify the most important factors affecting the van der Waals energy and non-bond energy.

From Figure 7 and Figure 8, a decrease in total potential energy when predicting non-bond and van der Waals energies for the FP_EB system compared to individual EB and FP systems can be attributed to several factors. One reason is the unique interaction between FP and EB molecules in the FP_EB system, resulting in more stable configurations with lower potential energy than the individual systems. Additionally, combining FP and EB molecules may lead to synergistic effects or enhanced binding interactions, further stabilizing the system and reducing its total potential energy. Furthermore, the combined system may experience fewer repulsive interactions or structural rearrangements, reducing total potential energy. In our previous work [32], we extensively investigated these potential reasons and comprehensively discussed the factors influencing the observed differences in total potential energy between the FP_EB system and individual EB and FP systems. Additionally, we conducted electronic computational analyses to elucidate the underlying mechanisms governing the energetics of the FP_EB system.

### 2.2. Total Potential Energy Prediction

In this initial scenario, we investigated the prediction performance of ensemble learning methods using three datasets. Each dataset consisted of nine input variables: total kinetic energy, bond energy, angle energy, torsion energy, inversion energy, non-bond energy, van der Waals energy, pressure, and temperature. Additionally, there was one output variable, total potential energy, in each dataset. The total number of data points in each dataset was 2000. The data were split into training and testing sets, with 80% of the data allocated for training and the remaining 20% for testing. The hyperparameters for the four models (Gradient Boosting, XGBoost, CatBoost, and Random Forest) were determined during the training phase using the training datasets. For Gradient Boosting (GB), the hyperparameters were set as follows: the number of trees = learning rate = 0.1, maximum depth of individual trees = 3, and the minimum number of samples required to split a node = 2. In XGBoost, the hyperparameters were configured with the following values: the number of trees = 100, learning rate = 0.3, and the maximum depth of individual trees = 6. Similarly, for CatBoost, the hyperparameters were set as follows: the number of trees = 100, learning rate = 0.3, and the maximum depth of individual trees = 6. Lastly, for Random Forest, the hyperparameters were established with the number of trees = 100 and a minimum number of samples required to split a node = 5. These hyperparameters were chosen to optimize the performance of each model based on the characteristics of the training data.

Predicting the TPE for EB, FP, and FP_EB adsorption states offers several significant advantages. However, TPE serves as a fundamental indicator of the overall stability and energetics of molecular systems. By accurately predicting TPE, we gain valuable insights into the thermodynamic behavior and equilibrium states of these systems. This understanding is crucial for optimizing molecular designs and predicting their performance under different conditions. In this study, TPE plays a key role in determining the interactions between molecules and their environment, including phosphorene surfaces. Therefore, the precise prediction of TPE enables us to assess the binding affinities and interaction strengths of antiviral drug molecules with phosphorene, which is essential for designing effective drug delivery platforms. Furthermore, TPE prediction facilitates the exploration of energy landscapes and potential energy surfaces, aiding in identifying stable conformations and reaction pathways. Overall, the accurate prediction of TPE enhances our ability to comprehend molecular systems’ underlying energetics and structural properties, thereby guiding the rational design and optimization of pharmaceutical agents and nanomaterial-based drug delivery systems.

Table 1 presents a summary of the evaluation metrics, including RMSE, MAE, and the $R^{2}$, computed from the testing datasets for EB, FP, and FP_EB. The results demonstrate the effectiveness of the investigated ensemble learning models (i.e., Gradient Boosting, XGBoost, CatBoost, and RF) in predicting the TPE. For the EB scenario, all models demonstrate high prediction accuracy, as evidenced by low RMSE and MAE values, along with high $R^{2}$ scores close to one. This indicates that the models are able to closely approximate the TPE values, capturing the underlying patterns and variations in the data. Similarly, for the FP scenario, the models exhibit excellent predictive performance, with consistently low RMSE and MAE values and high $R^{2}$ scores. This suggests that the models are effective in predicting TPE even when the antiviral drug is changed to FP. However, under the FP_EB scenario, the prediction performance slightly decreases for all models compared to the other scenarios. While the models still achieve relatively low RMSE and MAE values, the $R^{2}$ scores are slightly lower, indicating a lesser degree of variance explained by the models. This can be attributed to the combined effect of both FP and EB, which introduces additional complexity to the prediction task. From Table 1, the Catboost shows superior RMSE performance under FP_EB compared to EB data. This discrepancy may stem from several factors. Firstly, EB data tend to exhibit greater variance than FP_EB data, as depicted in Figure 1. Moreover, FP_EB compound data may demonstrate clearer patterns and relationships, which Catboost effectively captures, leading to lower RMSE values. Additionally, differences in the distribution and characteristics of input features between the two compounds can further influence prediction quality across the models.

**Figure 9:**
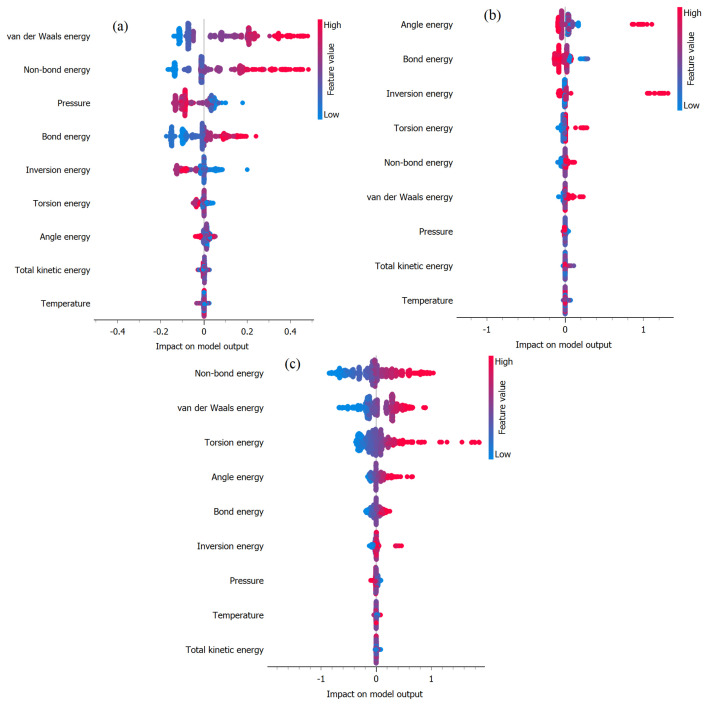
Identification of important variables impacting total potential energy prediction using mean SHAP values under (**a**) EB, (**b**) FP, and (**c**) FP_EB.

Figure 9a–c illustrates SHAP plots generated using the XGBoost methodology to identify important variables impacting total potential energy prediction. These plots utilize mean SHAP (SHapley Additive exPlanations) values to quantify the influence of each input variable on the model’s predictions under (a) EB, (b) FP, and (c) FP_EB. SHAP provides valuable insights into the importance of features by quantifying the contribution of each variable to individual predictions [33,34,35]. By analyzing these plots, we can discern the relative significance of different input features in determining the total potential energy, thereby aiding in the interpretation and understanding of the model’s decision-making process. In the SHAP plot, the x-axis represents the SHAP values, indicating the impact of each feature on the model’s output prediction. Features with positive SHAP values contribute to increasing the prediction, while those with negative SHAP values contribute to decreasing it. The y-axis lists the input features, with each feature represented by a vertical bar. The color intensities of the bars correspond to the feature value: darker shades indicate higher feature values, while lighter shades indicate lower values. By examining the length and direction of the bars, we can assess the magnitude and direction of each feature’s influence on the prediction. Features with longer bars have a greater impact on the prediction, while those with shorter bars have a lesser impact. Additionally, the horizontal line in the middle of the plot represents the baseline prediction, and the individual points represent the SHAP values for each feature in the dataset.

In Figure 9a, the analysis underscores the crucial role of van der Waals energy in influencing TPE prediction in the EB scenario. Van der Waals energy emerges as the most influential variable, signifying that interactions governed by van der Waals forces exert the most substantial impact on TPE prediction. This underscores the significance of spatial arrangements and interactions between molecules, particularly dispersion forces, in stabilizing the system. Additionally, non-bond energy ranks as the second most influential factor in TPE prediction for EB. Conversely, variables such as pressure, bond energy, and torsion energy exhibit lower mean SHAP values, indicating their relatively minor contribution to TPE prediction. While these variables still play a role in shaping the energy landscape, their significance is overshadowed by the dominance of van der Waals interactions. These insights are essential for understanding the specific properties of molecular structures, compositions, orbitals, and electronic spatial conditions [29]. Figure 9b illustrates that under FP, no single factor appears to exert a significant dominance over others in influencing the prediction of TEP. From Figure 9b, it is evident that non-bond energy, van der Waals energy, and tension energy are the most influential factors impacting the prediction of TPE under FP_EB. These variables exhibit higher SHAP values, indicating their significant contribution to the overall prediction outcome. The prominence of non-bond energy and van der Waals energy underscores the importance of intermolecular interactions and bonding patterns in determining the total potential energy within the FP_EB system. Additionally, the influence of tension energy suggests the role of structural stability and conformational changes in modulating energy dynamics.

### 2.3. Van Der Waals Energy Prediction

In the second scenario, we aimed to predict the van der Waals energy for EB, FP, and FP_EB systems using ten variables, including total kinetic energy, bond energy, angle energy, torsion energy, inversion energy, non-bond energy, TPE, pressure, and temperature. The van der Waals energy is crucial, as it provides insights into the non-covalent interactions between molecules, which play a significant role in determining the stability and structural conformation of molecular systems. By accurately predicting van der Waals energy, we can better understand the intermolecular forces governing the behavior of antiviral drug molecules on phosphorene surfaces, aiding in the design and optimization of drug delivery systems. Table 2 presents the prediction quality of van der Waals energy using four ensemble models for EB, FP, and FP_EB based on testing data. The results demonstrate that all four models offer high-quality predictions across the three datasets, as evidenced by their high $R^{2}$ values close to one and low RMSE and MAE scores. This indicates that the models effectively capture the complex interactions governing van der Waals energy in each system. Additionally, leveraging these ensemble models enhances the robustness and reliability of the predictions, providing valuable insights into the molecular interactions essential for drug design and optimization.

**Figure 10:**
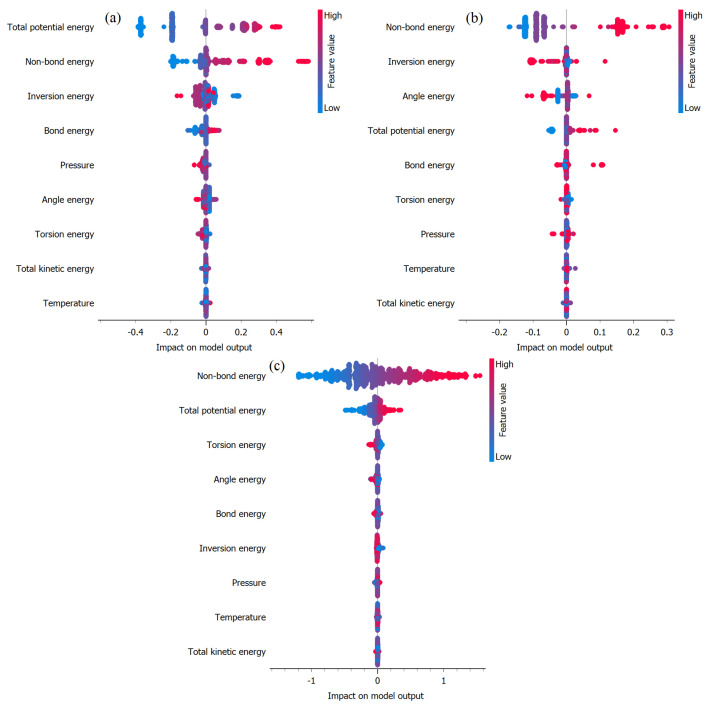
Identification of important variables impacting van der Waals energy prediction using mean SHAP values under (**a**) EB, (**b**) FP, and (**c**) FP_EB.

Figure 10a–c presents the SHAP plots aimed at identifying the significant variables influencing van der Waals energy prediction under (a) EB, (b) FP, and (c) FP_EB. Remarkably, non-bond energy emerges as the most impactful factor affecting the prediction of van der Waals energy across all three scenarios. This observation highlights the crucial role of non-covalent interactions and molecular configurations in determining the van der Waals energy within each system. Additionally, the SHAP plots provide valuable insights into the relative contributions of various input variables. This helps us gain a deeper understanding of the fundamental mechanisms that alter energy dynamics.

### 2.4. Non-Bond Energy Prediction

The last scenario aimed to predict the non-bond energy for EB, FP, and FP_EB. Predicting non-bond energy for EB, FP, and FP_EB systems is crucial for understanding molecular interactions, optimizing drug design, and improving therapeutic outcomes. It provides insights into intermolecular forces, guiding the development of more effective antiviral drugs with enhanced stability and binding affinity. Table 3 outlines the prediction quality of non-bond energy using four ensemble models for EB, FP, and FP_EB based on testing data. Across all three datasets, the models exhibit high-quality predictions, as indicated by their $R^{2}$ values approaching one and low RMSE and MAE scores. This shows how well the models are able to capture the complex dynamics that alter non-bond energy in each system.

**Figure 11:**
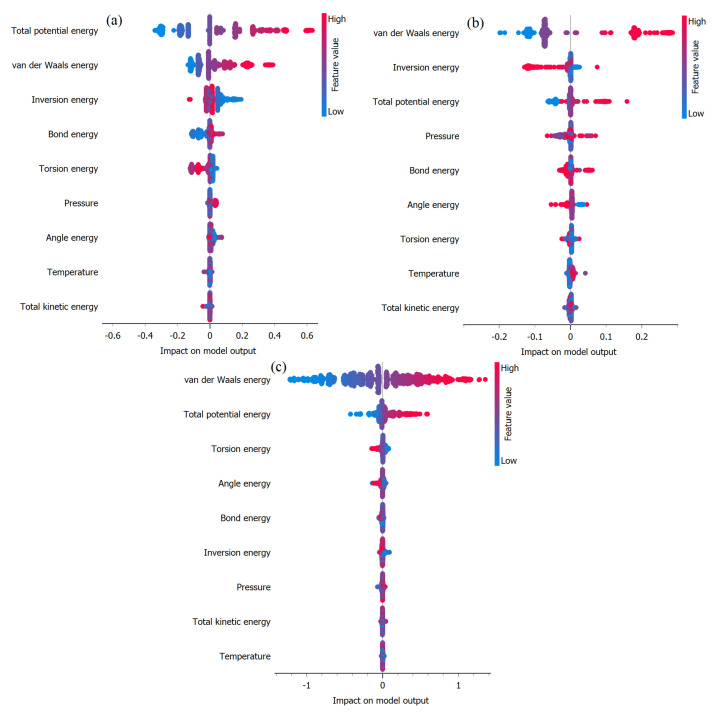
Identification of important variables impacting non-bond energy prediction using mean SHAP values under (**a**) EB, (**b**) FP, and (**c**) FP_EB.

Figure 11a–c depicts the SHAP plots used to identify the significant variables that have a notable impact on the prediction of non-bond energy. These plots are based on mean SHAP values across (a) the EB configuration, (b) the FP configuration, and (c) the FP_EB adsorption states. In the EB configuration, the TPE emerges as the most influential variable for predicting non-bond energy, closely followed by van der Waals energy. Conversely, in the FP and FP_EB scenarios, van der Waals energy assumes a dominant role as the primary factor impacting non-bond energy prediction. This observation suggests that the intermolecular interactions controlled by van der Waals forces play a critical role in comprehending the non-bond energy within these systems. Finally, the distinct trends in variable importance emphasize the unique characteristics and underlying mechanisms that regulate the Hamiltonian in each specific configuration.

### 2.5. Density Functional Calculations

In this section, we employed density functional theory (DFT) combined with previously employed molecular dynamics (MD) and machine learning (ML) techniques to explore the optimal spatial arrangement of FP, EB, and hybrid FP_EB molecules on the nanocarrier. Our aim is to determine the preferred adsorption sites of FP, EB, and FP_EB molecules on the BP nanocarrier, specifically in relation to aromatic molecules and 2D biomaterials at their respective ground states. The present study investigates the interaction between favipiravir (C_5_H_4_FN_3_O_2_) and ebselen (C_13_H_9_NOSe) molecules with black phosphorus (BP) by focusing on the most energetically stable configurations. To identify the most stable non-bond energy or interaction energy, the BP surface is divided into a grid with dimensions of (1 × 1) and grid spacing of 0.25 Å. The interaction of the molecules at each grid site is thoroughly examined through structural relaxation at a temperature of T = 0 K. Subsequently, the configuration that corresponds to the lowest interaction energy geometry is chosen. It is common for multiple favorable sites to be available for drug adsorption onto two-dimensional (2D) surfaces. However, in this study, only the geometry with the lowest energy level is retained for further analysis.

The non-bond energy, $\Delta E_{\mathrm{Non}-\mathrm{Bond}}$, for the drugs on the surface is determined by the following equation:

(1) $$\Delta E_{\mathrm{Non}-\mathrm{Bond}}=E_{\mathrm{Tot}}-\left(E_{\mathrm{BP}}+E_{\mathrm{Mol}}\right).$$

Here, $E_{\mathrm{BP}}$ represents the unrelaxed energy of the surface, $E_{\mathrm{Mol}}$ represents the unrelaxed isolated energy of the molecule, and $E_{\mathrm{Tot}}$ represents the relaxed energy of the surface with the adsorbed molecule. A negative value of $E_{\mathrm{Non}-\mathrm{Bond}}$ indicates stable adsorption. Throughout the entire manuscript, we focus solely on presenting the numerical values of the non-bond energy for the sake of simplicity. It should be noted that our calculations are specifically focused on characterizing the interaction of the molecules with the respective surface. As a result, the reported non-bond energies are expressed in units of kcal/mol per molecule. The comprehensive details of the interaction between these drugs and black phosphorus are provided in the main paper.

**Figure 12:**
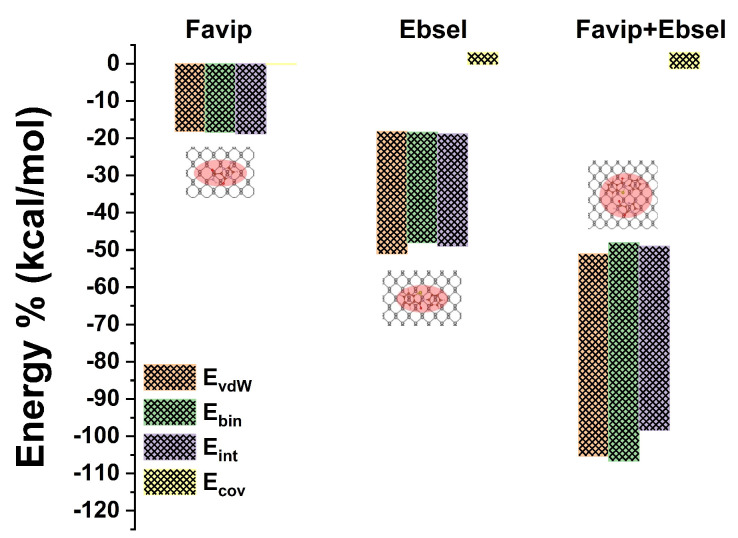
The van der Waals, binding, interaction, and covalent energies associated with the antiviral molecule on the BP slab are examined in the context of favipiravir, ebselen, and the combined favipiravir+ebselen hybrid drug.

The phosphorene refers to the monolayer form of black phosphorus, which is the most thermodynamically stable geometry of phosphorus. It has an orthorhombic lattice structure and consists of puckered layers of atoms [29,32]. The molecular orientation of PB was analyzed by studying the binding, formation, interaction, and covalent energies of complex structures with different molecular orientations. Various energetically favorable physisorption geometries of polycyclic molecules on pristine BP were identified, as illustrated in Figure 12. Specifically, FP, EB, and FP_EB molecules aligned themselves along a zigzag row on the BP surface. However, the C_5_H_4_FN_3_O_2_ molecule exhibited stability due to the presence of carboxamide (–CONH_2_) moieties and the positioning of the pyrazine rings on top of the BP. In the case of the C_13_H_9_NOSe anchor, the molecule was stabilized by a central di-benzene ring on the BP surface, while the carboxyl and selenazol moieties remained on the top of the hollow sites. Finally, the FP_EB hybrid molecules exhibited a similar planar preferred mode, adjacent to the BP monolayer, and were coupled through van der Waals interactions, similar to the FP and EB physisorbed alone on a pristine BP surface.

**Figure 13:**
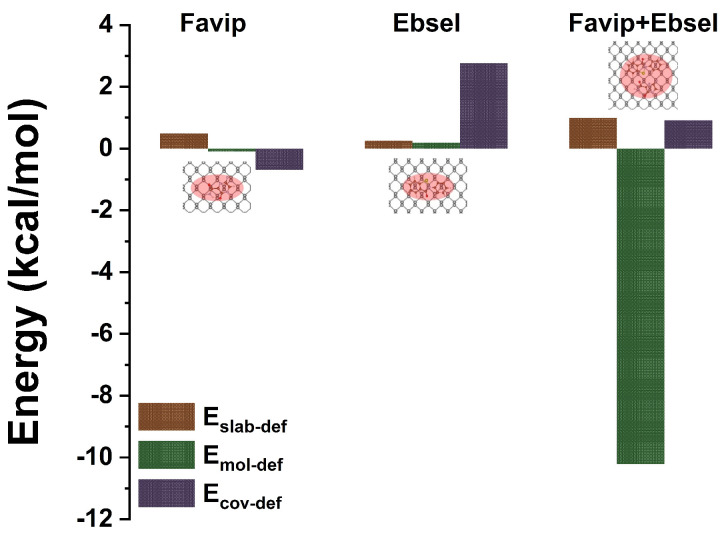
Deformation energies of the antiviral molecule on the BP slab, drug, and covalently bonded structures are examined for favipiravir, ebselen, and the combined favipiravir+ebselen hybrid drug.

The energy-minimized geometries of FP, EB, and FP_EB molecules interacting with a BP sheet, along with key distances, are presented in Table 4. The results indicate that the C_5_H_4_FN_3_O_2_ molecule adopts a parallel orientation because of its favorable energetic state, whereas both C_13_H_9_NOSe and hybrid molecules exhibit a planar orientation with respect to BP surface. This can be attributed to the strong vdW interaction between the carboxamide and selenium moieties with the P atom, respectively. The calculated vertical heights between FP, EB, and FP_EB molecules and BP are approximately 3.19 Å, 3.27 Å, and 3.23 Å, respectively. Moreover, in Figure 12, the binding energies for FP, EB, and FP_EB are −18.45 kcal/mol, −19.75 kcal/mol, and −18.80 kcal/mol, respectively. Notably, in the case of FP_EB molecules, there is a tendency for the simultaneous interaction between the two terminal functions and BP monolayer to be optimized, resulting in a contraction of the vdW forces over the BP surface. This understanding can be derived from the decomposition of the interaction energy into pure GGA and van der Waals dispersion energy terms, which predominantly influence all configurations ($E_{GGAHyb}$ = −1.38 kcal/mol and $E_{vdwHyb}$ = −14.42 kcal/mol). The interaction energy of FP_EB (−19.58 kcal/mol) is significantly higher compared to those of FP (−18.91 kcal/mol) and EB (−10.21 kcal/mol) because of the affinity of oxygen and selenium atoms with the lone pair of phosphorus atoms in FP_EB [29]. This leads to a pull on the phosphorene sheet toward the -CONH_2_ and -CONSe moieties in FP, EB, and hybrid molecules. Additionally, the energy decomposition analysis (Table 4) reveals that the binding energy consists of destabilized (positive) and minor deformation energies (FP, EB, and BP sheet), as well as stabilized (negative) interaction energies between FP, EB, and FP_EB with BP (vdW interactions). The deformation energies of the BP sheet, calculated from the optimal deformed BP nanocarrier corresponding to the adsorption structures, are relatively weak for FP, EB, and hybrid molecules. However, the deformation energy of hybrid FP_EB molecules (−10.15 kcal/mol) is more stabilized, similar to those of FP and EB molecules (refer to Figure 13).

## 3. Materials and Methods

This section highlights the use of molecular dynamics simulations alongside ensemble learning models in our study. Machine learning techniques are employed to accelerate density functional theory (DFT) relaxations, leading to faster convergence. These methods effectively capture the interaction energies of drug molecules, such as FP, EB, and their hybrids, when adsorbed on a BP single layer. The integration of machine learning with traditional approaches represents a significant advancement in understanding drug interactions with ultra-thin film crystalline 2D materials.

### 3.1. Molecular Dynamics Simulations

Molecular dynamics (MD) simulations, based on atomic force fields, offer a precise means to replicate the non-covalent geometries of diverse molecular systems spanning from non-equilibrium states to thermodynamic configurations at the atomic scale. In the present investigation, the data presented herein were acquired through the application of molecular dynamics simulations, implemented utilizing Forcite software (BIOVIA Material studio, https://www.3ds.com/products/biovia/materials-studio, accessed on 27 March 2024) [28].

To achieve the appropriate adsorption of favipiravir (FP) and ebselen (EB) drugs onto a monolayer of black phosphorus (BP), we adopted a (5 × 4) supercell model of black phosphorus, encompassing 80 phosphorus atoms for the nanocarrier. A vacuum region, extending 20 Å perpendicularly to the BP film, was introduced to mitigate any spurious periodic effects. Our simulation incorporated a Hamiltonian comprising kinetic energy, potential energy, interaction energy, and free energy, with temperature and pressure variables as parameters. The simulation was conducted at 350 K and atmospheric pressure. We employed an NVT canonical ensemble regulated by a Nosé thermostat. During the MD simulations, we used a time step of 0.3 fs. The simulations were carried out for a total of 100 ps, allowing us to explore a range of computational states and achieve the desired thermodynamic conditions for accurate MD calculations.

### 3.2. Ensemble Learning Techniques

This study investigates four powerful ensemble learning methods: Random Forest, Gradient Boosting, XGBoost, and CatBoost. Each algorithm is outlined in the subsequent subsections. The main key of this technique is to compare their performance and assess their effectiveness in predicting three key energy parameters: 1. Total potential energy, 2. van der Waals energy, and 3. non-bond energy related to ebselen (EB), favipiravir (FP), and the hybrid FP_EB case.

#### 3.2.1. Random Forest

Random Forest is an ensemble learning technique that uses a collection of decision trees to enhance overall accuracy and robustness, introduced by Breiman in 2001 [36]. It can be used for regression and classification tasks. In the case of regression, the algorithm is used to predict continuous values rather than class labels. Particularly effective in handling complex relationships and noisy datasets, Random Forest constructs an ensemble of decision trees trained independently on random data subsets, mitigating overfitting and improving stability. Randomness is introduced through bootstrap sampling and random feature selection during tree construction. Prediction aggregation, where the predictions from individual trees are averaged, results in a more accurate and stable final output. The algorithm’s strengths lie in its high predictive accuracy, robustness to outliers, and the ability to handle complex relationships in noisy datasets [37]. Moreover, Random Forest provides insights into feature importance, aiding in the interpretation of the model. Essential considerations include tuning parameters, like the number of trees, maximum depth of trees, minimum samples for split and leaf, and feature subset size. In summary, Random Forest for regression proves to be a powerful and flexible tool, widely applicable in various domains where precise predictions of continuous variables are essential [38].

#### 3.2.2. Gradient Boosting

Gradient Boosting stands as a potent machine-learning technique adept at handling both regression and classification tasks with finesse. Introduced as an ensemble learning method, it builds a strong predictive model by sequentially combining the outputs of multiple weak learners, often decision trees. Unlike Random Forest, Gradient Boosting focuses on correcting errors made by the previous models in the ensemble [39]. The GB process involves fitting a weak learner to the ensemble’s residuals (the differences between the actual and predicted values). The term “gradient” in Gradient Boosting refers to optimizing the loss function gradient, steering the models toward minimizing residuals through gradient descent [40]. Noteworthy for its high predictive accuracy, Gradient Boosting handles complex relationships in data effectively. Weak learners, often shallow decision trees, contribute sequentially to the ensemble, preventing overfitting and ensuring simplicity. The learning rate parameter governs the contribution of each weak learner, influencing model complexity. Like Random Forest, Gradient Boosting provides insights into feature importance, aiding in understanding variable impact. It thrives on medium-sized datasets and demonstrates robustness to different data types. Parameters such as the number of trees, learning rate, tree depth, and subsample require careful tuning for optimal performance. In summary, Gradient Boosting proves to be a powerful and versatile ensemble method, widely employed for its precision in capturing intricate patterns and relationships in both regression and classification tasks. Gradient boosting is closely linked to the gradient descent process. Let $\hat{y}\left[i\right]$ be a group of predictors, initially derived from the data points y[i] in a dataset of size *n*. The loss function L(Θ), often representing half the sum of squared residuals, is defined as [41]

(2) $$L\left(\Theta \right)=\frac{1}{2}\sum_{i=1}^{n}{\left(y\left[i\right]-\hat{y}\left[i\right]\right)}^{2}$$

To minimize the error, an adjustment of $\hat{y}\left[i\right]$ is performed by computing the gradient, denoted as ∇L(Θ):

(3) $${\hat{y}}_{\mathrm{new}}=\nabla L\left(\Theta \right)\left(y,{\hat{y}}_{\mathrm{old}}\right)$$

Subsequently, $\hat{y}\left[i\right]$ is updated with a marginal value, μf[i], determined by the gradient of the remaining variables:

(4) $${\hat{y}}_{\mathrm{new}}={\hat{y}}_{\mathrm{old}}+\mu f\left[i\right]$$

Here, μf[i] represents the computed gradient at each step to adjust the estimator, minimizing the pseudo-residual error.

### 3.3. XGBoost

XGBoost addresses ranking, classification, and regression challenges across diverse datasets. It decomposes the objective function obj(Θ) into a universally differentiable training loss function L(Θ) applicable to all regression trees and distinct regularization terms for each individual regression tree denoted as Ω(Θ) (Chen and Guestrin, 2016):

(5) obj(Θ)=L(Θ)+Ω(Θ)

Choosing a suitable loss function is contingent upon the characteristics of the dataset. Nevertheless, the regularization term in obj(Θ) is contingent upon two loss functions, the count of leaves, and additional constants, as indicated in Equation (6) (Chen and Guestrin, 2016):

(6) $$\Omega \left(\Theta \right)=\alpha L_{1}\left(\Theta \right)+\frac{1}{2}\lambda L_{2}\left(\Theta \right)+\gamma Q$$

Here, $L_{1}\left(\Theta \right)$ and $L_{2}\left(\Theta \right)$ represent loss functions, *Q* stands for the number of leaves, and (α,λ,andγ) denote constants specific to XGBoost that impart a conservative nature to the model. The construction of trees involves a greedy function, calculating gain to facilitate the determination of optimal split decisions. In XGBoost, decision trees are concurrently built in a multi-level manner, organizing tree attributes once in each stage [41]. To gain a more thorough understanding of the XGBoost algorithm, it is recommended to review the details presented in [42].

#### CatBoost

CatBoost, developed by Yandex, is a powerful gradient-boosting library designed to handle categorical features efficiently. Its standout feature lies in its ability to internally manage the conversion of categorical variables into numerical representations during training, alleviating the need for extensive preprocessing [43]. The algorithm utilizes a symmetric tree structure, optimizing training speed without the necessity for column reordering [44]. CatBoost incorporates regularization techniques to enhance model robustness, including depth regularization and learning rate annealing [45]. Particularly user-friendly, CatBoost offers effective default parameters, reducing the complexity of hyperparameter tuning. In regression tasks, CatBoost excels in minimizing mean squared error, constructing an ensemble of decision trees that iteratively correct errors made by the ensemble so far [46]. Its efficient handling of categorical features and robust performance make CatBoost a valuable choice for regression modeling, especially in scenarios where categorical features play a significant role [47].

Table 5 provides a concise overview of the characteristics of the investigated ensemble learning models.

### 3.4. Key Steps in the Proposed Framework

**Figure 14:**
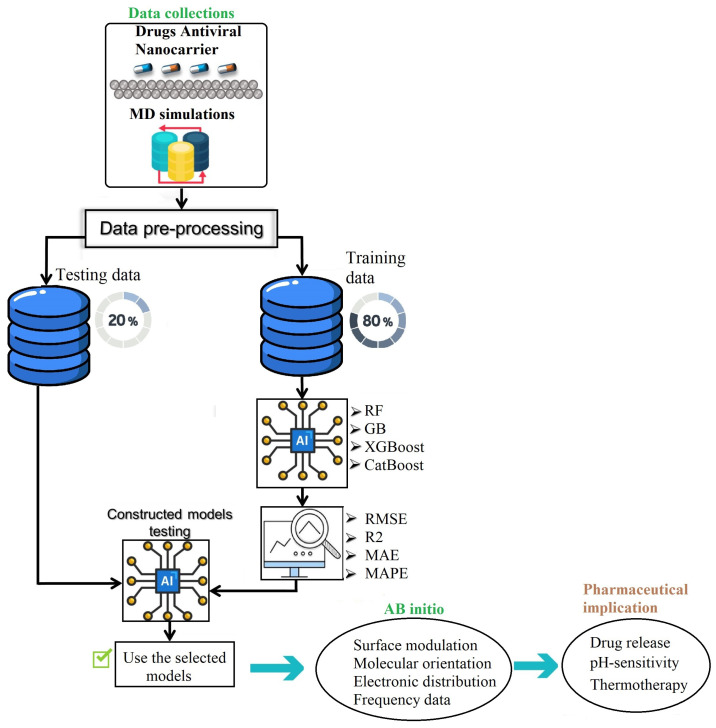
Representation of the applied predictive framework and the core processes in the exploration of antiviral drugs for small molecules on black phosphorus. This involves developing quantitative structure–activity relationship models with the objective of optimizing primary structures for pharmaceutical applications.

This study explores the applicability of ensemble learning techniques, namely, Random Forest, Gradient Boosting, XGBoost, and CatBoost, in predicting three crucial energy parameters—total potential energy, van der Waals Energy, and non-bond energy—associated with ebselen (EB), favipiravir (FP), and their combination, FP_EB, derived from molecular dynamics (MD) simulations. The prediction methodology, illustrated in Figure 14, comprises two stages: training and testing. Initially, 80% of the data is utilized to train the machine learning models, incorporating a five-fold cross-validation approach. Subsequently, the trained models are employed to predict the three energy parameters using the testing data. The evaluation of predictions is performed using metrics such as mean absolute error (MAE), root mean squared error (RMSE), and R-squared ($R^{2}$).

RMSE measures the average deviation of predicted values from actual values. It is calculated as

(7) $$\mathrm{RMSE}=\sqrt{\frac{1}{n}\sum_{i=1}^{n}{\left(y_{i}-{\hat{y}}_{i}\right)}^{2}},$$

MAE is another measure of the average deviation between predicted values and actual values. It is calculated as

(8) $$\mathrm{MAE}=\frac{1}{n}\sum_{i=1}^{n}\left|y_{i}-{\hat{y}}_{i}\right|,$$

$R^{2}$ is a statistical measure that represents the proportion of the variance in the dependent variable that is predictable from the independent variables. It is calculated as

(9) $$R^{2}=1-\frac{\sum_{i=1}^{n}{\left(y_{i}-\hat{y}i\right)}^{2}}{\sum {i=1}^{n}{\left(y_{i}-\bar{y}\right)}^{2}},$$

where $y_{i}$ represents the actual values, $\hat{y}$ represents the predicted values, $\bar{y}$ represents the mean of the actual values, and *n* is the number of observations.

### 3.5. Density Functional Calculations

Density functional theory (DFT) is a widely used approach in the field of quantum chemistry, offering the ability to compute various properties of atomistic systems, including molecules, crystals, surfaces, interfaces, and biomaterial carriers, when combined with machine learning. In this study, all DFT calculations were conducted using the Vienna Ab Initio Simulation Package (VASP 5.4) [48,49]. The electron–ion interactions were modeled using the projected augmented wave (PAW) formalism [50]. The exchange-correlation functional contribution to the total energy was determined using the generalized gradient approximation (GGA), specifically the modified Perdew–Burke–Ernzerhof (PBE) approach [50]. To accurately account for van der Waals (vdW) interactions, the D3-BJ approach, as established by Grimme et al. [51,52], was incorporated into all calculations [53,54]. A (5 × 4) supercell model of a black-phosphorus monolayer comprising 80 phosphorus atoms was utilized as the nanocarrier for the adsorption of the FP and EB drugs. To mitigate any unphysical periodic effects, a vacuum region of 20 Å was introduced perpendicular to the BP film. Considering the large size of the supercell, numerical integration was performed over the Brillouin zone using a 3 × 3 × 1 Γ-centered k-point grid [55]. For electronic and frequency calculations, a denser 5 × 5 × 1 mesh was employed during the self-consistent field (SCF) procedure. The pseudo-wave functions were expanded using a plane-wave basis set with an energy cut-off of 450 eV. To ensure accuracy, an energy convergence criterium of 1 × 10^−5^ eV was adopted. Structural relaxation was carried out using the conjugate gradient technique until the force on each atom was below 0.01 eV/Å. This methodology has previously proven successful in describing fullerene adsorption on single-layer graphene [6].

## 4. Conclusions

This study presents a novel methodology integrating molecular dynamics simulations with machine learning (ML) techniques to precisely estimate Hamiltonian parameters, including potential energy, van der Waals energy, and non-bond energy, among others. The approach involves training an ML model to discern the correlation between various structural configurations and their respective Hamiltonian parameters using simulated antiviral molecules on a BP monolayer surface. The subsequent application of the trained ML model enables the prediction of critical parameters for an unexplored experimental structure, with corresponding structural attributes determined through density functional theory (DFT) computations. Moreover, our exploration of feature importance using SHAP plots provided valuable interpretability, elucidating the contribution of individual variables to the predictive models. The effectiveness of this methodology is substantiated by its ability to faithfully replicate the adsorption configurations derived from density functional theory calculations while accurately forecasting potential energy, van der Waals energy, and non-bond energy. These results highlight the promise of our proposed approach as a viable avenue for achieving robust and precise parameter estimation crucial for drug discovery endeavors. In future research, there are several ways to make our models better. We can incorporate more sophisticated machine learning algorithms, such as deep learning architectures, to make predictions that are more accurate and reliable. This can help us understand how drug molecules bind to 2D nanomaterials properly. We can also look at more drugs and molecular systems to obtain an advanced understanding of how they work. Additionally, we can study how environmental factors like temperature and pH affect how drugs bind to low-dimensional materials. This can help us understand how effective drugs are in real-world situations. Finally, these suggested avenues for future research will contribute to advancing our understanding of drug-binding mechanisms, improving predictive models, and ultimately facilitating the development of more effective and reliable pharmaceutical therapies.

## Figures and Tables

**Table 1:** Prediction quality of the total potential energy using the four ensemble models.

	**EB**		
Methods	RMSE	MAE	$R^{2}$
Gradient Boosting	0.022	0.002	0.998
XGBoost	0.02	0.002	0.998
CatBoost	0.035	0.007	0.995
Random Forest	0.019	0.002	0.998
	**FP **		
Methods	RMSE	MAE	$R^{2}$
Gradient Boosting	0.018	0.001	0.997
XGBoost	0.014	0.001	0.998
CatBoost	0.015	0.004	0.998
Random Forest	0.019	0.001	0.997
	**FP_EB**		
Methods	RMSE	MAE	$R^{2}$
Gradient Boosting	0.094	0.045	0.981
XGBoost	0.118	0.027	0.97
CatBoost	0.078	0.032	0.987
Random Forest	0.15	0.045	0.951

**Table 2:** Prediction quality of van der Waals energy using the four ensemble models.

	**EB**		
Methods	RMSE	MAE	$R^{2}$
Gradient Boosting	0.0065	0.0003	0.9998
XGBoost	0.0152	0.0008	0.9989
CatBoost	0.0183	0.0031	0.9984
Random Forest	0.0048	0.0002	0.9999
	**FP**		
Methods	RMSE	MAE	$R^{2}$
Gradient Boosting	0.0083	0.0005	0.9984
XGBoost	0.0071	0.0004	0.9989
CatBoost	0.0133	0.0023	0.9959
Random Forest	0.0101	0.0009	0.9976
	**FP_EB**		
Methods	RMSE	MAE	$R^{2}$
Random Forest	0.0163	0.0034	0.9994
Gradient Boosting	0.0105	0.0046	0.9998
XGBoost	0.0139	0.0054	0.9996
CatBoost	0.0406	0.0228	0.9964

**Table 3:** Prediction quality of non-bond energy using the four ensemble models.

	**EB**		
Methods	RMSE	MAE	$R^{2}$
Gradient Boosting	0.0072	0.0003	0.9997
XGBoost	0.0140	0.0008	0.9990
CatBoost	0.0187	0.0032	0.9983
Random Forest	0.0054	0.0002	0.9999
	**FP**		
Methods	RMSE	MAE	$R^{2}$
Gradient Boosting	0.0087	0.0005	0.9983
XGBoost	0.0071	0.0004	0.9988
CatBoost	0.0119	0.0019	0.9967
Random Forest	0.0118	0.0010	0.9968
	**FP_EB**		
Methods	RMSE	MAE	$R^{2}$
Gradient Boosting	0.0105	0.0046	0.9998
XGBoost	0.0141	0.0058	0.9996
CatBoost	0.0407	0.0227	0.9964
Random Forest	0.0130	0.0030	0.9996

**Table 4:** Adsorption distances d–d (in Å), non–bond energy and van der Waals energy predictions using ML and PBE-D3 for FP_EB, FP, and EB adsorption states (kcal/mol) at T = 0 K.

	FP_EB	FP	EB
Distance d−d (Å)	3.19	3.27	3.23
${\mathrm{ML}}_{Non-Bond}$ (kcal/mol)	−16.03	−16.00	−19.00
PBE–D3*_Non−Bond_* (kcal/mol)	−19.60	−18.45	−10.12
${\mathrm{ML}}_{vdW}$ (kcal/mol)	−11.89	−17.30	−10.67
PBE–D3*_vdW_* (kcal/mol)	−14.42	−18.22	−12.98

**Table 5:** Comparative analysis of ensemble learning models

Model	Key Concepts	Advantages	Disadvantages
RF	Ensemble of decision trees; uses bootstrap sampling and random feature selection; predictions aggregated through averaging.	High predictive accuracy; robust to outliers; handles complex relationships and noisy datasets; provides insights into feature importance.	Computationally expensive for large datasets and many trees; may not perform well on highly imbalanced datasets.
GB	Sequential model building; minimizes residuals through gradient descent optimization; combines weak learners to form a strong model.	High predictive accuracy; robust to different types of data; provides insights into feature importance; effective on medium-sized datasets.	Prone to overfitting if not properly tuned; sensitive to noisy data; requires careful parameter tuning.
XGBoost	Extreme Gradient Boosting; handles categorical features efficiently; incorporates L1 and L2 regularization; parallel and distributed computing support.	Exceptional predictive performance efficient handling of categorical features robust to overfitting; effective default parameters; suitable for large datasets.	May require careful tuning of hyperparameters; relatively complex algorithm learning curve for beginners.
CatBoost	Handles categorical features without extensive preprocessing; utilizes symmetric tree structure; incorporates depth regularization; effective default parameters.	Efficient handling of categorical features; robust to overfitting; effective default parameters; user-friendly; suitable for regression tasks.	May be computationally intensive for very large datasets; learning curve for beginners due to limited documentation; limited interpretability compared to simpler models.

## Data Availability

The data will be available on request.
